# Application of a Hybrid Model of Big Data and BP Network on Fault Diagnosis Strategy for Microgrid

**DOI:** 10.1155/2022/1554422

**Published:** 2022-03-25

**Authors:** Chundi Jiang, Zhiliang Xia

**Affiliations:** ^1^Logistics Engineering College, Shanghai Maritime University, Shanghai 201306, China; ^2^Electrical and Information Engineering, Quzhou University, Quzhou 324000, China; ^3^Wenzhou Polytechnic, Wenzhou 325035, China

## Abstract

Aiming at the characteristics of timely transmission, rapid update, and large magnitude of microgrid data, based on the large data samples generated by microgrid operation, a fault diagnosis and analysis method of microgrid systems supported by big data is proposed in this paper. The multisource joint feature vectors of microgrid are extracted using Wavelet transform, Rayleigh entropy, and big data technology, which combine short-circuit current and voltage. The extracted feature dataset is clustered and segmented to realize deep data mining. Combining BP neural network and big data, the fault diagnosis of microgrid is realized. The simulation results show that the BP neural network algorithm based on big data support can accurately identify the type and phase of internal faults in microgrid, which is more suitable for extracting the temporal characteristics of information and spatiotemporal correlation of data to realize the prediction of big data and solve the core problems in the analysis of big data of microgrid faults, and the accuracy is as high as 96.8%.

## 1. Introduction

Big data refers to the dataset that cannot be captured, managed, and processed by conventional software tools within a certain period of time. It is a massive and diversified information asset of high growth rate that needs a new processing mode to have stronger decision-making power, insight, and process optimization ability. “Volume, Variety, Value, Velocity, and Veracity” are the “5V” characteristics of big data. Relevant research and surveys have pointed out that the global annual data growth rate is basically twice or even higher than that of the previous year. In the next 10 years, nonstructural data will account for about 90% and the data patterns will be different. It will become impossible to analyze based on previous experience. Therefore, it is necessary to study relevant data mining technologies and understand and master the basic “5V” characteristics of big data, which is particularly important for data analysis based on big data. BP neural network can be applied to the prediction of models and the study of the relationship between different models. Therefore, the combination of big data technology and BP neural network can deal with large and complex nonlinear structural problems from a statistical point of view, with high stability and accuracy. Big data fault analysis refers to collecting a large amount of data through real-time analysis and mining of microgrid fault information to master the microgrid operation characteristics, accurately predict the microgrid topology behavior, and improve the capabilities of service and risk control. The key to big data is to be able to quickly obtain useful information from a large amount of data or quickly realize big data assets. Therefore, the information processing of big data is often based on cloud computing. Cloud computing is the product of a new era. Taking cloud computing as the development strategy, big data is applied to cloud computing to obtain a series of data that can be used for fault analysis, so as to form a big data fault diagnosis model.

The reasonable development and utilization of green and clean energy have become an important topic with increasingly prominent energy crisis, environmental pollution, and slow recovery caused by large power grids. Microgrid has been widely used because of its flexible installation locations, less pollution, and high energy efficiency. Its technical problems have also attracted the extensive attention of researchers at home and abroad. One of the important research fields is fault diagnosis technology, which guarantees safe and reliable operation. The common fault types of internal lines in the microgrid are single-phase grounding short circuit, two-phase short circuit, three-phase short circuit, etc. Conventional fault diagnosis methods cannot be directly applied to microgrid and work well due to the difference between current and voltage and bidirectional power flow. It is an urgent problem to find new fault diagnosis and identification methods for microgrid because of the changing characteristics of fault voltage and current.

A lot of research has been carried out and been applied in practice to the fault diagnosis technology of microgrid. The research mainly focuses on the following two aspects: Firstly, fault diagnosis is carried out according to the changes of circuit breakers, the flexible topology, protection elements, and other equipment status in the microgrid. For example, the voltage and bus current signals of the low-voltage circuit breaker are used for fault diagnosis. Using SOM neural network and multiagent systems, the simulation results show that good diagnosis results can be achieved for a single fault, but the situation of multiple faults in the same period needs further study [1, 2]. In [3], a Petri net analysis model using line fault protection information is established. The corresponding protection set information needs to be updated while the topology changes without remodeling, which can adapt to the variability of the microgrid topology structure. However, the identification accuracy of fault diagnosis will be affected when the protection device and circuit breaker have such factors as refusing to operate or misoperation.

Secondly, the abnormal change of voltage or current is used to diagnose the fault in the microgrid. The transient recovery performances of microgrid under different control modes are compared and analyzed by studying the change of current amplitude and the transient duration of overvoltage when a short-circuit fault occurs in the microgrid [4, 5]. According to the impedance and impedance angle, the symmetrical component method was used to analyze the voltage and current in the microgrid during a fault. However, it is difficult to accurately extract the fault component when the system is unstable, is oscillating, has interference, or has continuous fault. At the same time, its effect is a lack of experimental verification while only theoretical and methodological research is carried out.

The fault voltage and current signal are used to realize fault diagnosis and identification in a combined wavelet packet with a neural network, but the fault phase cannot be accurate to the line [6, 7]. The voltage signal of the optical storage microgrid line is analyzed to realize the safe and reliable operation of the optical storage microgrid based on genetic algorithm [8]. Another fault detection scheme based on deep neural networks and wavelet transform for microgrid was proposed in [9]. The authors in [10] employed an approach focusing on identifying and evaluating the faulted line section by implementing data mining and wavelet packet transform. At present, the scale of microgrid data has increased exponentially, and the traditional data processing methods cannot afford to process large-scale data. At the same time, the big data of the microgrid system runs through all nodes of smart grid, which is far beyond the scope of traditional power system monitoring. The current data processing platform is difficult to meet the requirements of smart grid for the processing of power system data. In particular, it is difficult to achieve real-time data, which results in the loss and repetition of power grid data. In the era of big data, how to process microgrid data to analyze and diagnose the working conditions of power equipment is an urgent task for the development of intelligent microgrids. In order to solve the problems of incomplete fault information, single information structure, and imperfect diagnosis results in the existing microgrid fault diagnosis methods, this paper extracts and reconstructs the features of multisource data using BP neural network based on big data technology, obtains the spatiotemporal correlation characteristics between different data, and then realizes fault prediction and diagnosis better.

The main contributions of this paper are as follows: First, most of the existing research literature is about the relationship between the amplitude of voltage, current, and fault, but less about the relationship between the time-frequency characteristics of voltage and current and the impact of time-frequency characteristics on fault. In fact, the time-frequency characteristics of voltage and current on the fault will have a greater impact. Secondly, this paper not only analyzes the relationship between voltage and current but also studies the regulatory role of voltage, current, and fault. Third, this paper combines BP neural network and big data to predict the spatiotemporal correlation of data in order to realize fault diagnosis using big data analysis. Considering that the voltage and current of three-phase lines contain rich transient sudden change signals when the internal lines of the microgrid fail, which can effectively reflect the fault characteristics, this paper obtained the high-frequency and low-frequency details of the signal using wavelet analysis, Rayleigh entropy, and other theoretical methods to extract the characteristic vectors of three-phase line fault voltage and current from massive microgrid data. Based on the analysis of fault information, the BP neural network is applied to fault diagnosis and identification. Trained with historical data as samples, the BP neural network model supported by big data is constructed to realize the accurate diagnosis of fault type and phase.

The rest of this paper is arranged as follows: Section 2 describes fault characteristics; Section 3 gives wavelet packet decomposition and energy entropy; Section 4 provides construction of the BP neural network fault diagnosis model; Section 5 describes fault diagnosis algorithm and process; Section 6 gives simulation analysis and experimental verification; Section 7 concludes the study.

## 2. Fault Characteristics Analysis

### 2.1. Microgrid Prototype

**Figure 1:**
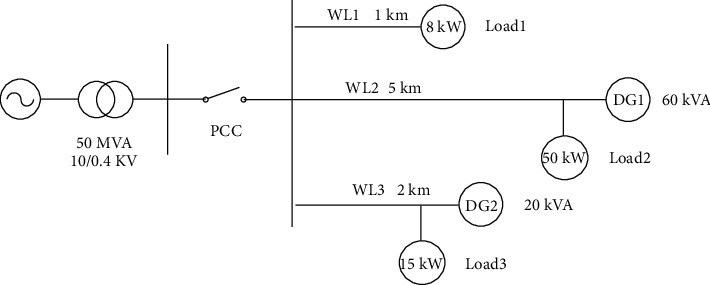
System structure.

A microgrid prototype is shown in Figure 1. The bus voltage is 220 V and connected to the distribution network through the PCC of the common connection point. DG1 is a photovoltaic power generation unit with a capacity of 60 kVA. DG2 is a wind power source with a capacity of 20 kVA. There are three power transmission lines in the microgrid: WL1 is 1 km long with 8 kW load; WL2 is 5 km with 50 kW load; and WL3 is 2 km long with 15 kW load. The fault resistance and grounding resistance are both set as 0.001 Ω when single-phase faults happen, while the grounding resistance is set as 10 mΩ when two-phase or three-phase short circuits occur.

**Figure 2:**
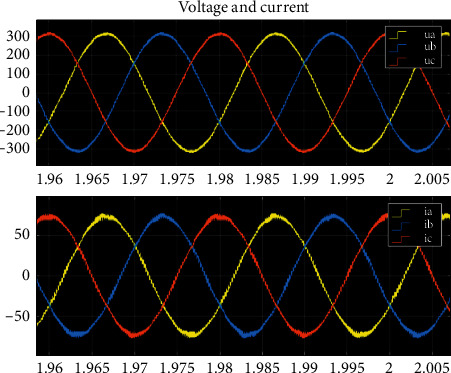
Three-phase output current waveform in normal operation.

The three-phase output voltage and current are symmetrical when the microgrid operates normally. The current waveform is shown in Figure 2. The three-phase output current is equal, and the phase difference is 120°.

### 2.2. Fault Analysis

**Figure 3:**
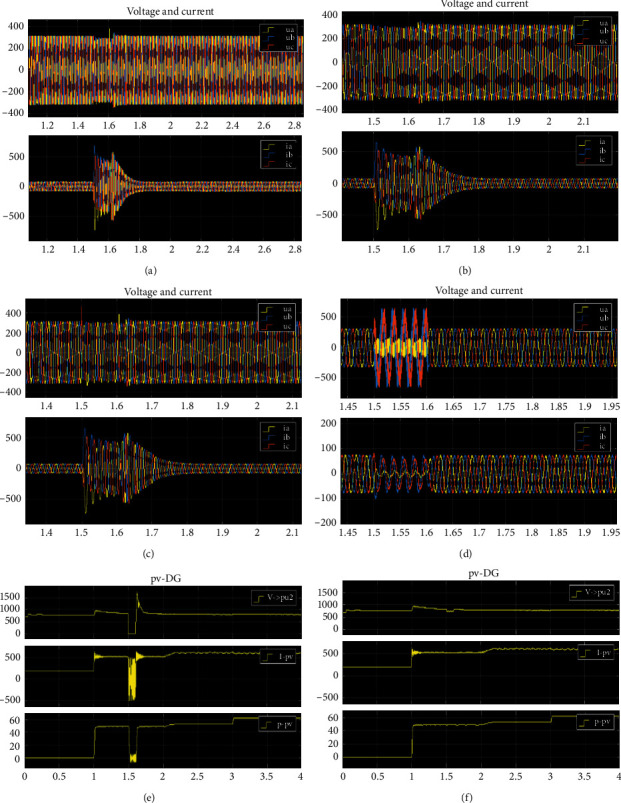
Partial fault voltage and current curves: (a) three-phase short circuit (1.5 s–1.6 s); (b) two-phase short circuit (1.5 s–1.6 s); (c) two-phase ground fault (1.5 s–1.6 s); (d) single-phase ground (1.5 s–1.6 s); (e) PV parameters of three-phase fault; (f) PV parameters of single-phase fault.

The Matlab simulation platform is used to analyze the variation of line voltage and current under different fault types and fault positions. In this paper, Matlab/Simulink 2018b software is mainly used in the process of modeling and simulation. Simulink is mainly responsible for the construction of the whole system and offline simulation verification. At the same time, it can be combined with StarSim HIL and StarSim RCP software of Yuankuan; then, the hardware in the loop test is realized. The simulation analysis is carried out at 10%, 50%, 70%, and 85% of the microgrid side, respectively, while single-phase ground fault, phase-to-phase short circuit, two-phase ground fault, or three-phase short-circuit fault happens. The simulation time is set as 0.5 s, and the fault will be removed in 0.4 s after the fault occurs in 0.1 s. When the fault occurs, the voltage and current at the fault point are shown in Figure 3.

Figure 3 shows that the voltage at the fault point decreases significantly after the short circuit, the amplitude of fault current increases, and there are certain components of harmonic and aperiodic components. It is very likely to cause microgrid instability and protection misoperation even after the fault is removed. The fault diagnosis method based on the single signal has certain limitations, so it is necessary to consider the voltage and current signals to build a new feature vector for fault diagnosis.

The fault data information during microgrid operation includes steady-state data, parameter data, alarm events, etc. The circuit information includes frequency, voltage, current, harmonic voltage, harmonic current, voltage imbalance, current imbalance, flicker, power and power factor in the circuit, power grid clutter interference, vibration, temperature and humidity, harmonic interference, abnormal events, and other indicators. Through the analysis and processing of these data, accurate fault characteristics can be extracted to realize fault identification and diagnosis.

## 3. Wavelet Packet Decomposition and Energy Entropy

### 3.1. Wavelet Packet Decomposition

The wavelet packet selects the optimal basis to decompose the original signal in the frequency domain, which improves the ability of signal analysis, avoids the defect of fixed time frequency of wavelet decomposition, and accurately reflects the nature and characteristics of the signal. It has good time-domain and frequency-domain positioning characteristics and excellent signal adaptability.

Let *ϕ*(*x*) be an orthogonal scaling function and *ψ*(*x*)be a wavelet function; then, the two-scale function equation is as follows:

(1) $$\begin{array}{c} \phi \left(\text{x}\right)=\sqrt{2}\underset{\text{k}}{\sum }{\text{h}}_{\text{k}}\phi \left(2\text{x}-\text{k}\right),\\ \psi \left(\text{x}\right)=\sqrt{2}\underset{\text{k}}{\sum }{\text{g}}_{\text{k}}\phi \left(2\text{x}-\text{k}\right), \end{array}$$

where h_k_ is the scale coefficient and g_k_ is the wavelet coefficient.

Defining a basis function u_0_(x)=*ϕ*(x) and u_1_(x)=*ψ*(x), then the two-scale equation is generalized as follows:

(2) $$\begin{array}{c} \left\{\begin{array}{c} {\text{u}}_{2\text{n}}\left(\text{t}\right)=\sqrt{2}\underset{\text{k}\in \text{z}}{\sum }{\text{h}}_{\text{k}}{\text{u}}_{\text{n}}\left(2\text{t}-\text{k}\right),\\ {\text{u}}_{2\text{n}+1}\left(\text{t}\right)=\sqrt{2}\underset{\text{k}\in \text{z}}{\sum }{\text{g}}_{\text{k}}{\text{u}}_{\text{n}}\left(2\text{t}-\text{k}\right), \end{array}\right. \end{array}$$

where the constructed sequence {u_n_(x)} is the wavelet packet of basis function u_0_(x)=*ϕ*(x).

The parameter *j* is the scale index, k is the position index, and *n* is the oscillation number; then,

(3) $$\begin{array}{c} \left\{\begin{array}{c} {\text{d}}_{k}^{\text{j},2\text{n}}=\underset{\iota \in \text{z}}{\sum }{\text{h}}_{\text{k}-2\iota }{\text{d}}_{\iota }^{\text{j}+1,\text{n}},\\ {\text{d}}_{k}^{\text{j},2\text{n}+1}=\underset{\iota \in \text{z}}{\sum }{\text{g}}_{\text{k}-2\iota }{\text{d}}_{\iota }^{\text{j}+1,\text{n}}. \end{array}\right. \end{array}$$

The wavelet packet reconstruction algorithm is as follows:

(4) $$\begin{array}{c} {\text{d}}_{k}^{\text{j}+1,\text{n}}=\underset{\iota \in \text{z}}{\sum }\left({\text{h}}_{\text{k}-2\iota }{\text{d}}_{\iota }^{\text{j},2\text{n}}+{\text{g}}_{\text{k}-2\iota }{\text{d}}_{\iota }^{\text{j},2\text{n}+1}\right). \end{array}$$

**Figure 4:**
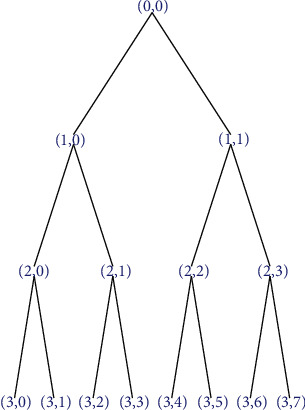
3-level wavelet packet decomposition structure.

Taking 3-level wavelet packet decomposition as an example, the process of wavelet packet decomposition and reconstruction is shown in Figure 4. Node (*i*, *j*) represents the *j*th node in layer *i* (*i* = 0,1,2,3; *j* = 0,1,2,…, 7), and each node represents a signal with certain characteristics. For example, the node (0,0) represents the original signal S, node (1,0) represents the low-frequency coefficient of the first layer of wavelet packet decomposition, and node (1,1) represents the high-frequency coefficient of the first layer.

The relationship between the original signal S and its decomposition coefficient is as follows:

(5) $$\begin{array}{c} \text{S}=\text{S}\left(3,0\right)+\text{S}\left(3,1\right)+\ldots +\text{S}\left(3,7\right). \end{array}$$

### 3.2. Feature Entropy Extraction and Multisource Joint Eigenvector Construction

Entropy can measure the degree of information uncertainty in information theory, such as information entropy and relative entropy [11, 12]. The Shannon entropy extracted from the wavelet packet decomposition and reconstruction can reflect the high-frequency and low-frequency characteristics of the signal more accurately and has stronger anti-interference performance [6]. The time complexity of the frequency component of the signal transient can be accurately expressed by the singular entropy of the Rayleigh wavelet packet, which is more conducive to the identification and diagnosis of fault signals.

Let the random signal  X={x_0_, x_1_,…, x_N−1_, x_N_}; the probability of occurrence of x_i_ is

(6) $$\begin{array}{c} {\text{p}}_{\text{i}}=\text{p}\left({\text{x}}_{\text{i}}\right),\\ \text{i}=0,1,2,\ldots \text{n}-1,\\ \overset{\text{N}}{\underset{\text{i}=0}{\sum }}{\text{p}}_{\text{i}}=1. \end{array}$$

Then, the Rayleigh entropy of *X* is

(7) $$\begin{array}{c} {\text{H}}_{\text{q}}\left(\text{X}\right)=\frac{1}{1-\text{q}}\text{ln}\overset{\text{N}}{\underset{\text{i}=1}{\sum }}{\text{p}}_{p}^{q}. \end{array}$$

The expression of energy entropy fused with wavelet packet decomposition is

(8) $$\begin{array}{c} E_{i,j}=\frac{1}{1-q}ln\underset{j}{\sum }{\text{p}}_{\text{i},\text{j}}^{\text{q}}. \end{array}$$

The vector *T* is composed of the energy entropy of each frequency band:

(9) $$\begin{array}{c} \text{T}=\left[{\text{E}}_{\text{i},0},{\text{E}}_{\text{i},1},{\text{E}}_{\text{i},2}\ldots {\text{E}}_{\text{i},2^{\text{n}}-1}\right]. \end{array}$$

To facilitate signal analysis, the vector *T* can be normalized and represented by T′ when the energy entropy of each frequency band is relatively large.

(10) $$\begin{array}{c} \text{E}=\overset{2^{\text{n}}-1}{\underset{\text{j}=0}{\sum }}{\text{E}}_{\text{i},\text{j }},\\ {\text{T}}^{\text{'}}=\frac{\text{T}}{E}=\left[\frac{E_{i,0}}{\text{E}},\frac{E_{i,1}}{\text{E}},\frac{E_{i,2}}{\text{E}}\ldots \frac{E_{i,2^{n}-1}}{\text{E}}\right]. \end{array}$$

After feature entropy extraction, the voltage and current signals are fused by the interval crossing method to form a new multisource fault feature vector for fault diagnosis. The final signal eigenvector is obtained.

## 4. Construction of the BP Neural Network Fault Diagnosis Model

In 1986, the BP neural network was proposed by Rumelhart and McClelland, which has become one of the most popular neural network models because of its strong functions of self-learning and adaptability. The BP network can solve the fault diagnosis problem of some complex systems and provide theoretical research and technical implementation methods for more intelligent diagnosis methods.

**Figure 5:**
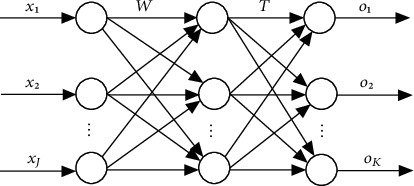
Topological structure of the BP neural network.

With the support of big data technology, the development of the BP neural network gradually tends to mature. When analyzing and diagnosing power grid faults, it can convert relay protection information into model input and take the possible faults of microgrid as an output. On this basis, it completes the selection of the sample set. Then, it cleans and segments the sample, compares the difference between the actual output and the expected output, realizes the layer-by-layer adjustment of the weight and threshold, and stops after repeated times, and the difference is consistent with the standard. The BP neural network is mainly composed of an input layer, a hidden layer, and an output layer. The topological structure of a typical three-layer BP neural network is shown in Figure 5.

Regulation: *J* is the number of nodes in the input layer, the node serial number is *j*, input vector $\dot{X}=\left[{\text{x}}_{1},{\text{x}}_{2}\ldots {\text{x}}_{\text{j}}\ldots {\text{x}}_{\text{J}}\right]$,  j=1,2 … J, K is the number of nodes in the output layer, node serial number is *k*, $\dot{O}=\left[{\text{o}}_{1},{\text{o}}_{2}\ldots {\text{o}}_{\text{k}}\ldots {\text{o}}_{\text{K}}\right]$ is the output vector, k=1,2 … K, *L* is the number of nodes in the hidden layer, the node serial number is *ι*, W_j*ι*_ represents the connection weight between the jth neuron in the input layer and the *ι* neuron in the hidden layer, T_*ι*k_ represents the connection weight between the *ι*th neuron in the hidden layer and the kth output neuron in the output layer, and the input of the *ι*th node in the hidden layer is I_*ι*_. The output is *y*_*ι*_. The activation function is f(·). Then, the input and output of the *ι*th neuron are expressed as

(11) $$\begin{array}{c} {\text{I}}_{\iota }=\overset{\text{J}}{\underset{\text{j}=1}{\sum }}{\text{W}}_{\text{j}\iota }{\text{x}}_{\text{j}},\\ y_{\iota }=\text{f}\left({\text{I}}_{\iota }\right). \end{array}$$

Given N samples $\left({\dot{X}}_{\text{n}},{\dot{\text{d}}}_{\text{n}}\right)$ (*n* = 1, 2,…, N), the expected output vector of the input vector ${\dot{X}}_{\text{n}}$ is ${\dot{\text{d}}}_{\text{n}}$. The objective function is defined as the sum of error squares between the expected output and the actual output during backpropagation.

(12) $$\begin{array}{c} {\text{E}}_{\text{n}}=\frac{1}{2}\overset{\text{K}}{\underset{\text{k}=1}{\sum }}{\left[{\text{d}}_{\text{n}}\left(\text{k}\right)-{\text{O}}_{\text{n}}\left(\text{k}\right)\right]}^{2}. \end{array}$$

The total error of N samples is defined as

(13) $$\begin{array}{c} \text{E}=\frac{1}{2\text{N}}\overset{\text{N}}{\underset{\text{n}=1}{\sum }}\overset{\text{K}}{\underset{\text{k}=1}{\sum }}{\left[{\text{d}}_{\text{n}}\left(\text{k}\right)-{\text{O}}_{\text{n}}\left(\text{k}\right)\right]}^{2}. \end{array}$$

By adjusting the connection weight and threshold, the total error *E* is minimized and the weight changes along the negative gradient direction of the error function.

(14) $$\begin{array}{c} {\text{W}}_{\text{j}\iota }\left(\text{t}+1\right)={\text{W}}_{\text{j}\iota }\left(\text{t}\right)-\eta \frac{\partial \text{E}}{\partial {\text{W}}_{\text{j}\iota }}, \end{array}$$

where *t* is the number of iterations and *η* is the step size.

## 5. Fault Diagnosis Algorithm and Process

**Figure 6:**
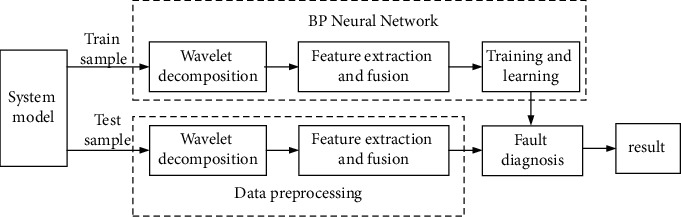
The flow chart of microgrid fault diagnosis.

Based on the algorithm of the first two sections, a new method of short-circuit fault diagnosis for microgrid is proposed in this paper. The fault feature is extracted by wavelet packet decomposition, and the energy entropy is calculated. The multisource joint eigenvector is composed of voltage and current characteristic entropy, which is used as the BP network input and realizes fault diagnosis. Firstly, the time-frequency analysis of the three-phase current and voltage signals is carried out by wavelet packet decomposition. Then, the Shannon energy entropy is calculated as the signal feature vector, and the multisource joint eigenvector is composed of cross fusion and used as the input of the BP neural network for training and learning. The fault type and phase of microgrid can be accurately identified and diagnosed. The algorithm flow is shown in Figure 6.

500 groups of line fault sample data are randomly selected. Firstly, wavelet packet decomposition is used to analyze the signal time frequency, and then, Shannon energy entropy is used to extract the signal feature vector as the input signal of the BP neural network. The sample size for neural network training and learning is 80%. Another group of 10% data is used for verification, and the third group of 10% data is used for testing.

Using the BP neural network algorithm model, the multilayer feedforward network is trained according to the error backpropagation algorithm of microgrid fault output data, and then, through a large amount of sample learning, the input-output mode mapping relationship of the fault is stored, which can realize real-time and online mapping of fault information, making the complex nonlinear relationship in the output data samples become obvious. The accuracy of fault diagnosis is greatly improved, and the data error rate is reduced.

## 6. Simulation Analysis and Experimental Verification

### 6.1. Wavelet Packet Decomposition of Fault Current in Microgrid

According to the experimental analysis and comparison, the frequency band distribution of 2-level wavelet packet decomposition is too wide and the resolution is low, while those of the 4-level wavelet packet decomposition and 3-level wavelet packet decomposition are the same, but the amount of calculation is significantly increased. Therefore, this paper chooses 3-level wavelet packet decomposition for the signal.

**Figure 7:**
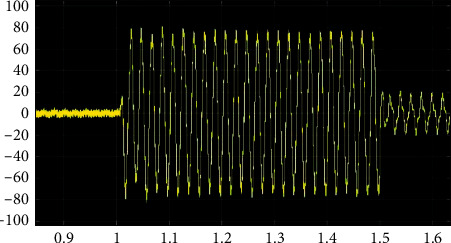
Short-circuit current of a single-phase earth fault.

As an example, the step of extracting the wavelet packet energy entropy of the A-phase current signal is illustrated by the A-phase single-phase grounding short-circuit current of line WL3 away from the microgrid side. The waveform diagram of the single-phase grounding short-circuit current is shown in Figure 7.

**Figure 8:**
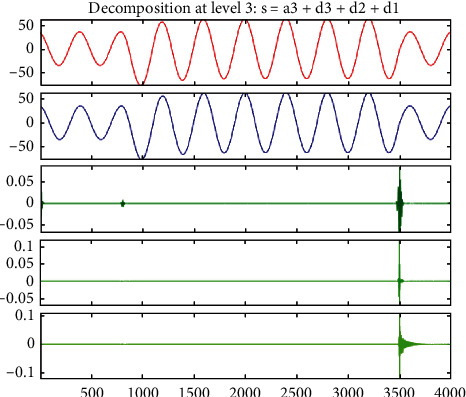
Wavelet packet decomposition of a single-phase grounding short-circuit current.

Comparing Figures 7 and 5, it can be found that when the system is short-circuited, the current changes suddenly and has transient fault information. The db6 wavelet base is selected to further extract the effective fault information. The A-phase current signal is decomposed by 3-level wavelet multiresolution using formula (2), which can obtain the wavelet packet decomposition coefficient and wavelet reconstruction signal. The current decomposition signal is shown in Figure 8.

It can be seen from the detailed diagram that the first impulse current wave is received at 800 sampling points and the second impulse current wave is received at 3500 sampling points. Considering the period, it can be used as the basis for the fault location of microgrid.

The wavelet packet reconstruction of the short-circuit current and voltage wavelet signal shows that the fault signal contains rich nonstationary fault signal components, so the wavelet packet reconstruction signal will immediately have obvious fluctuations at the time of fault, which can be used as an important criterion to judge whether the fault occurs in the internal lines of the microgrid and to calculate the wavelet energy entropy value. The fault types and fault phases of the internal lines of the microgrid can be well prepared using the BP neural network.

### 6.2. Extraction and Construction of Multisource Joint Eigenvector

The energy entropy of 8 wavelet reconstruction signals of A-phase short-circuit current is calculated using Shannon entropy formula (8), and then, an eigenvector *E* is formed as follows:

(15) $$\begin{array}{c} \text{E}={\left[{\text{E}}_{30},{\text{E}}_{31},{\text{E}}_{32},{\text{E}}_{33},{\text{E}}_{34},{\text{E}}_{35},{\text{E}}_{36},{\text{E}}_{37}\right]}^{\text{T}}, \end{array}$$

where E_30_, E_31_, E_32_, E_33_, E_34_, E_35_, E_36_, E_37_ is the entropy of wavelet reconstruction signals. Because the wavelet packet Shannon entropy can detect small abnormal changes in the signal, when the signal-to-noise ratio is low, it can extract the effective weak signal and eliminate the noise very well. The smaller the entropy value is, the more orderly the signal is, and vice versa. It has little influence on the accuracy of judgment with the amount of calculation being multiplied in the process of fault diagnosis and recognition using BP neural network; at the same time, the high-frequency signal component is more than one order of magnitude smaller than the low-frequency signal component, so E_30_, E_31_, E_32_, E_33_ can be taken as the input of BP neural network.

(16) $$\begin{array}{c} \text{E}={\left[{\text{E}}_{30},{\text{E}}_{31},{\text{E}}_{32},{\text{E}}_{33}\right]}^{\text{T}}. \end{array}$$

**Figure 9:**
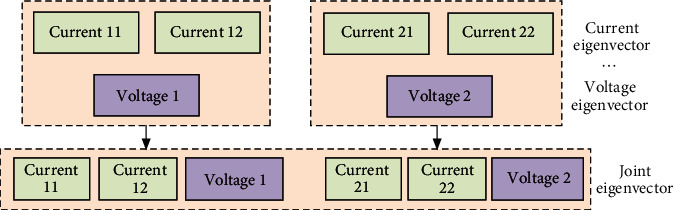
Association diagram of the joint fault feature vector.

In the same way, the other two-phase current and voltage signals are processed and 16-dimensional wavelet energy entropies are obtained. The multisource fault information is fused by the interval cross mode to form the feature vector for fault diagnosis. The fusion mode is shown in Figure 9.

Some multisource feature eigenvectors are shown in Table 1.

**Figure 10:**
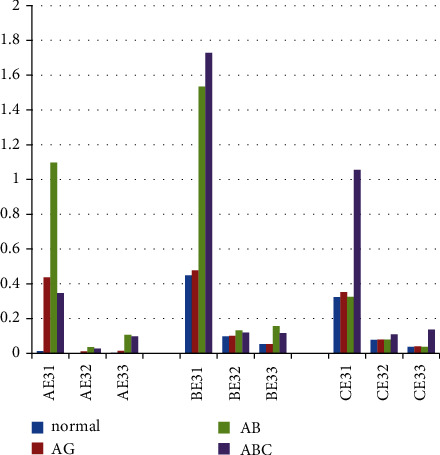
Energy entropy comparison.

Compared with the normal state energy entropy, some results are shown in Figure 10.

The energy entropy E_31_, E_32_, E_33_ of phase A is surely increased when A phase shows ground fault but B and C phases almost remain unchanged; the energy entropy E_31_, E_32_, E_33_ of two phases A and B are significantly increased when two-phase short circuit or two-phase grounding short circuit occurs; the energy entropy E_31_, E_32_, E_33_ of three phases are all improved when A, B, and C three-phase short-circuit occurs. This can be used as a basis for fault type identification and fault phase judgment in microgrid.

### 6.3. Training Results of BP Neural Network

**Figure 11:**
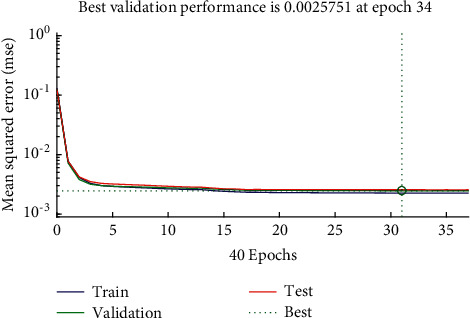
Training curve.

Three-phase current and voltage eigenvalues constitute a multisource fault eigenvector which is taken as the input vector of the BP neural network. The fault signal of microgrid is decomposed and reconstructed with a wavelet, and the number of input neuron nodes is set as 16. The state of the three phases and the neutral line are taken as output vectors, so the number of output neuron nodes of the neural network is set as 4. The output value of 1 indicates that the corresponding line is at fault or the fault phase is grounded, and 0 indicates that the corresponding line has no fault. The number of hidden layer neurons affects the training results of the model. The training accuracy is poor if too few nodes are selected; the training time and step size are relatively large when there are too many nodes, and it is easy to fit. This is verified by experiments, and combined with the empirical formula $\text{ L}=\sqrt{J+K}+\alpha$, this paper chooses 10 hidden layer nodes. The BP network stops the model training and learning when the training error meets the given requirements. The training curve is shown in Figure 11.

**Figure 12:**
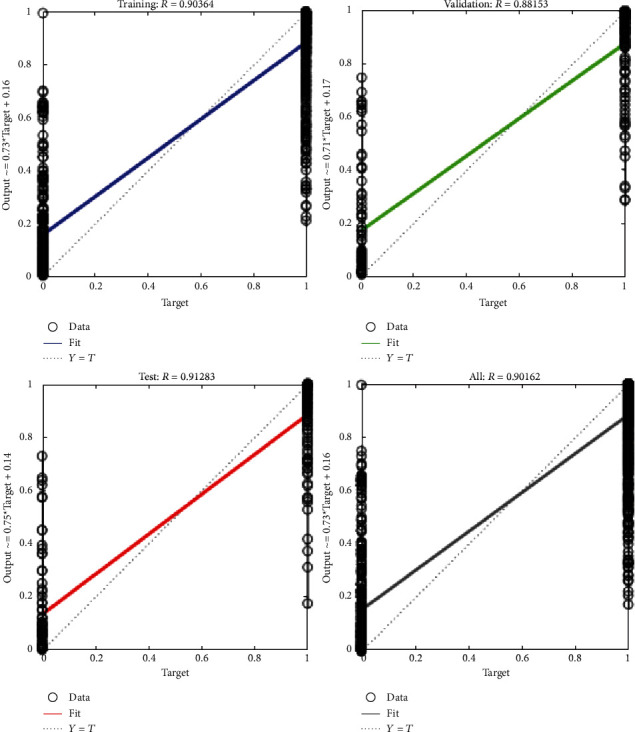
Fitting curve.

The fitting degree curve of neural network training is shown in Figure 12. Through observation, the fitting degree of neural network is high, the fitting degree of training and testing is more than 0.9, and the fitting degree of verification is also 0.88. Thus, BP neural network can accurately diagnose and identify faults.

### 6.4. Hardware in the Loop Simulation Test

**Figure 13:**
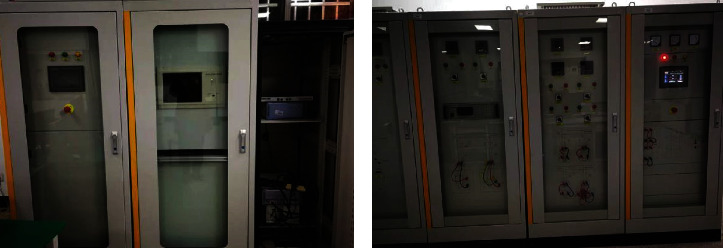
The hardware of the loop platform.

**Figure 14:**
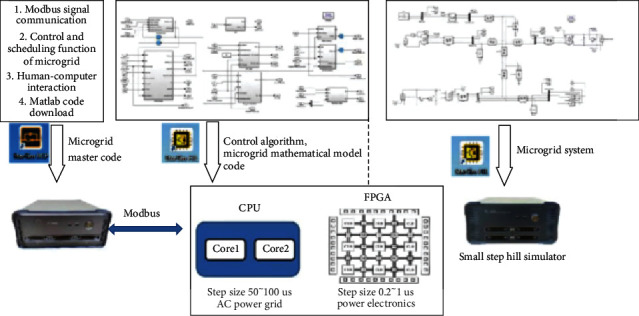
The system framework.

**Figure 15:**
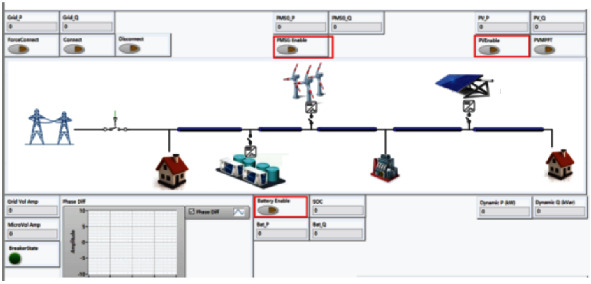
The microgrid interface.

Because the actual microgrid systems are built outdoors, where the natural conditions are bad and the system structure is complex, including a large number of power electronic device load units, the voltage level is high and dangerous. However, the construction of a traditional electrical laboratory has high construction costs and site requirements. It is difficult to simulate and research some special conditions such as faults considering safety and equipment maintenance. Therefore, the hardware in the loop simulation technology is applied to the experimental environment based on the existing equipment and conditions of the laboratory. The hardware in the loop simulation platform is composed of PXI hardware and StarSim HIL and StarSim RCP software developed by Yuankuan energy to realize microgrid fault identification and diagnosis. The hardware in the loop platform is shown in Figure 13, the system framework is shown in Figure 14, and the microgrid interface is shown in Figure 15.

### 6.5. Fault Diagnosis and Identification

The multisource feature vectors of microgrid test samples are brought into the trained BP neural network to diagnose and identify the fault type and phase. The actual output signal of the test sample is compared with the expected output signal. Some test samples and their fault diagnosis results are shown in Table 2.

The BP neural network method based on big data analysis extracts and abstracts the features of fault data and strengthens the spatiotemporal correlation of heterogeneous data to realize the prediction of fault type and phase. At the same time, the BP neural network method based on big data obtains accurate data information by constructing many invisible models and performing a large amount of data analysis and training. It can be seen from Table 2 that the trained BP neural network model can accurately and effectively identify the fault type and fault phase and the error between the actual output and the expected output value meets the requirements of fault diagnosis. The test results show that two samples have fault diagnosis among 50 samples; the accuracy rate reaches 96%, which meets the requirements for intelligent fault diagnosis of microgrid lines.

## 7. Conclusions

Aiming at the characteristics of real-time transmission, fast updating, and large-scale fault information data of microgrid, a microgrid fault diagnosis and analysis technology supported by big data is proposed in this paper. This technology combines Rayleigh entropy, wavelet packet decomposition method, and BP neural network to extract the fault feature vector of microgrid. The BP neural network method based on big data strengthens the spatiotemporal correlation of heterogeneous data to realize the prediction of fault type and phase. The BP neural network method based on big data obtains accurate results by constructing many invisible models and a large amount of data training. The experimental results show that the fault diagnosis and analysis technology based on big data support proposed in this paper has an accuracy of 96%, which fully meets the needs of engineering practical application. However, due to the complex topology and many fault types of microgrid, only five line fault types are considered in this paper. Therefore, in the next step, the technical method proposed in this paper needs to be applied to other fault diagnosis to verify the universality of the technical method proposed in this paper.

## Figures and Tables

**Table 1:** Some multisource feature eigenvectors.

Fault type	Fault position (%)	Feature eigenvector
AG	10	16.110, 4.3607, 0.0810, 0.1037, 23.616, 0.7045, 0.1075, 0.0562, 21.344, 0.5773, 0.0865, 0.0411, 56.320, 0.8069, 0.5211, 0.04385
AG	50	84.916, 0.4371, 0.0114, 0.0135, 22.398, 0.4777, 0.1000, 0.0537, 21.780, 0.3528, 0.0795, 0.0390, 125.102, 1.0972, 0.3917, 0.0846
ABG	10	17.934, 3.8371, 0.1305, 0.4891, 16.407, 3.3080, 0.2022, 0.1636, 15.357, 0.6490, 0.0887, 0.0665, 51.105, 0.9281, 0.5371, 0.0721
ABG	50	11.619, 1.2852, 0.0338, 0.1220, 10.578, 1.2189, 0.1338, 0.1893, 19.462, 0.4162, 0.0817, 0.0453, 59.219, 1.036, 0.9024, 0.06901
ABG	85	86.968, 0.2493, 0.0066, 0.0189, 80.033, 0.7853, 0.1145, 0.1464, 21.326, 0.3413, 0.0782, 0.0403, 129.264, 1.5973, 0.7941, 0.1026
AB	10	14.133, 4.2099, 0.0692, 0.1754, 14.353, 4.6465, 0.1665, 0.2262, 25.090, 0.3239, 0.0775, 0.0377, 22.683, 0.9251, 0.0529, 0.0904
AB	85	76.814, 1.0975, 0.0360, 0.1069, 76.832, 1.5341, 0.1328, 0.1570, 25.102, 0.3247, 0.0777, 0.0378, 110.469, 2.1963, 0, 9625, 0.0805
ABC	10	18.660, 1.1549, 0.0750, 0.4304, 17.224, 2.7162, 0.1434, 0.2369, 17.290, 2.7340, 0.1746, 0.5255, 59.132, 1.6938, 0.9934, 0.0982
ABC	70	10.779, 0.3454, 0.0274, 0.0971, 10.100, 1.7274, 0.1197, 0.1159, 10.127, 1.0555, 0.1089, 0.1373, 49.669, 1.0394, 0.6392, 0.0872
ABCG	10	18.615, 3.6540, 0.0415, 0.3515, 17.163, 3.3278, 0.1675, 0.3161, 17.579, 4.5462, 0.1377, 0.5702, 38.672, 0.9821, 0.0923, 0.0639
ABCG	70	10.766, 1.2417, 0.0176, 0.0644, 10.077, 1.9576, 0.1250, 0.1326, 10.210, 1.1433, 0.0915, 0.0571, 44.971, 0.7953, 0.0926, 0.0481
Normal		25.098, 0.0126, 0.0012, 0.0015, 25.088, 0.4485, 0.0973, 0.0522, 25.090, 0.3239, 0.0775, 0.0377, 64.925, 1.0369, 0.9835, 0.0485

**Table 2:** Part of the test samples and fault diagnosis results.

Fault type	Expected output	Test sample	Actual output
AG	1, 0, 0, 1	60.968, 0.062, 0.007, 0.034, 22.730, 0.451, 0.098, 0.054, 22.653, 0.327, 0.078, 0.039, 89.361, 1.804, 0.9735, 0.0639	0.973, 0.017, 0.015, 0.987
AC	1, 0, 1, 0	14.410, 1.223, 0.076, 0.414, 25.088, 0.449, 0.097, 0.052, 14.183, 1.532, 0.151, 0.450, 49.035, 1.329, 0.405, 0.0579	0.998, 0.027, 1.045, 0.028
ACG	1, 0, 1, 1	91.732, 1.770, 0.041, 0.104, 20.424, 0.516, 0.099, 0.056, 96.708, 1.272, 0.089, 0.071, 120.488, 1.409, 0.1088, 0.053	1.009, 0.025, 0.998, 1.012

## Data Availability

The datasets used to support the findings of this study are available from the corresponding author upon request.

## References

[1] Rajendra K. P., Kumar S. V. Multi Agent System Based Intelligent Fault Diagnosis with Fault Current Limiter for Microgrid.

[2] Lu Q., Ye Y. Z., Jiang C. D. (2017). Fault diagnosis of microgrid system based on wavelet singular entropy and SOM neural network. *Journal of Shandong University*.

[3] Sun T. J., Qu L. P., Guan H. S. (2021). Power grid fault diagnosis based on intelligent optimization fuzzy Petri net. *Control Engineering*.

[4] Kang Q., Luo Y., Lu X. J., Gao P. (2019). Simulation of microgrid fault characteristics based on converter control strategy. *Power system protection and control*.

[5] Hu P. (2017). Analysis and Research on short circuit fault of distribution line in microgrid system. *Machinery Manufacturing*.

[6] Jiang C. D., Qiu L., Ye Y. Z. (2020). Short circuit fault diagnosis of microgrid based on Shannon entropy and BP neural network. *Southern Agricultural Machinery*.

[7] Yang Y. J., Chen Y., Yang K. (2017). Fault diagnosis of microgrid line based on wavelet packet analysis and neural network. *Automatica e Instrumentacion*.

[8] Yang Y. J., Mao F. (2018). Fault diagnosis of optical storage microgrid line based on RTDS and neural network. *Renewable Energy*.

[9] Liu S., Yang D. (2021). Identification and detection algorithm of electric energy disturbance in microgrid based on wavelet analysis and neural network. *EURASIP Journal on Wireless Communications and Networking*.

[10] Jamali S., Ranjbar S., Bahmanyar A. (2020). Identification of faulted line section in microgrids using data mining method based on feature discretisation. *International Transactions on Electrical Enegry Systems*.

[11] Jiang C. D., Yang W., Guo Y. (2018). Nonlocal means two dimensional histogram-based image segmentation via minimizing relative entropy. *Entropy*.

[12] Chen J. H., Gao Y. J., Jin Y. W. (2021). Microgrid fault diagnosis method based on MODWT and BP neural network. *Journal of Shanghai University of Electric Power*.

